# Retraction: Improving measles vaccine uptake rates in Nigeria: An RCT evaluating the impact of incentive sizes and reminder calls on vaccine uptake

**DOI:** 10.1371/journal.pone.0236542

**Published:** 2020-07-14

**Authors:** Steven Brownstone, Alison Connor, Daniel Stein, the PLOS ONE Editors

After this article [1] was published, concerns were raised about the author list, ethics approval, and study locations for the reported study.

Specifically:

- Concerns were raised that this article reports a study for which no collaborators in the local country are included in the author list or acknowledgments. The authors clarified that this was due to an error of omission. There were a number of Nigerian research and operational collaborators whose work was instrumental in conducting the study. The authors apologize for not having acknowledged these key contributors.
- Concerns were raised that the ethics approval was granted after the reported intervention dates. According to *PLOS ONE*’s policy on Human Subjects Research, studies involving human participants must have received approval from an institutional review board or ethics committee before the start of the study. The authors commented that they did not seek approval before the beginning of the trial because they did not originally view this work as research but rather, as a pilot experimentation to inform their partner organization’s operations. The authors argue that this is because the study did not involve primary data collection by the authors, it was based on data collected during the partner organization’s program operations, and in the authors’ view, was not originally intended to contribute to generalizable knowledge. Upon further consideration (specifically driven by the authors’ desire to use the results to contribute to generalizable knowledge), the authors sought ethical approval even though the pilot experimentation had already begun: the pilot study was included in an application to the National Health Research Ethics Committee of Nigeria (NHREC) along with a larger, forthcoming randomized control trial of their implementing partner’s program. The authors received the anonymized data from the partner organization only after the study received ethical approval.
- Questions were raised about apparent discrepancies in study location. The Methods section lists study locations of Nasarawa (in the North Central region), Anambra (in South East), and Akwa Ibom (in South South), whereas the study protocol (S1 Data file published with [1]) specifies study locations of the Northern states of Katsina, Zamfara, and Jigawa. The authors clarified that this paper [1] does not report the main clinical trial described in the protocol, but instead reports the pilot study conducted to inform incentive amounts for use in the main trial (see “Evidence to determine incentive amount” in the study protocol). As described in the protocol, the pilot study used data from nine clinics in Nasarawa, Anambra, and Akwa Ibom.

In light of the concerns about attribution and retrospective ethics approval, the authors and *PLOS ONE* Editors retract this article.

The *PLOS ONE* Editors apologize that these concerns were not addressed before the article was published.

The authors apologize for omitting acknowledgment of key contributors and for not working more closely with the Nigerian research community to co-produce this study. We also apologize for an error in judgement regarding the timing of the ethical application. At the time of retraction, the authors are working with the National Health Research Ethics Committee of Nigeria to resolve this issue.
